# Biomechanical Characteristics of Three Baseplate Rotational Arrangement Techniques in Total Knee Arthroplasty

**DOI:** 10.1155/2018/9641417

**Published:** 2018-06-06

**Authors:** KwanSu Kang, Young Woong Jang, Oui Sik Yoo, Dukyoung Jung, Sung-Jae Lee, Myung Chul Lee, Dohyung Lim

**Affiliations:** ^1^Department of Mechanical Engineering, Sejong University, Seoul, Republic of Korea; ^2^Central R&D Center, Corentec Co. Ltd., Seoul, Republic of Korea; ^3^Seongnam Senior Experience Complex, Eulji University, Seongnam, Republic of Korea; ^4^Department of Biomedical Engineering, Inje University, Gimhae, Republic of Korea; ^5^Department of Orthopedic Surgery, College of Medicine, Seoul National University, Seoul, Republic of Korea

## Abstract

**Introduction:**

Several ongoing studies aim to improve the survival rate following total knee arthroplasty (TKA), which is an effective orthopedic surgical approach for patients with severely painful knee joint diseases. Among the studied strategies, baseplate rotational arrangement techniques for TKA components have been suggested but have been the subject of only simple reliability evaluations. Therefore, this study sought to evaluate comparatively three different baseplate rotational arrangement techniques that are commonly used in a clinical context.

**Materials and Methods:**

Three-dimensional (3D) finite element (FE) models of the proximal tibia with TKA were developed and analyzed considering three baseplate rotational arrangement techniques (anterior cortex line, tibial tuberosity one-third line, and tibial tuberosity end line) for six activities of daily life (ADLs) among patients undergoing TKA. Mechanical tests based on the ASTM F1800 standard to validate the FE models were then performed using a universal testing machine. To evaluate differences in biomechanical characteristics according to baseplate rotational arrangement technique, the strain and peak von Mises stresses (PVMSs) were assessed.

**Results:**

The accuracy of the FE models used in this study was high (94.7 ± 5.6%). For the tibial tuberosity one-third line rotational arrangement technique, strains ≤ 50 *µ*strain (the critical bone damage strain, which may affect bone remodeling) accounted for approximately 2.2%–11.3% and PVMSs within the bone cement ranged from 19.4 to 29.2 MPa, in ADLs with high loading conditions. For the tibial tuberosity end line rotational arrangement, strains ≤ 50 *µ*strain accounted for approximately 2.3%–13.3% and PVMSs within the bone cement ranged from 13.5 to 26.7 MPa. For anterior cortex line rotational arrangement techniques, strains ≤50 *µ*strain accounted for approximately 10.6%–16.6% and PVMSs within the bone cement ranged from 11.6 to 21.7 MPa.

**Conclusion:**

The results show that the most recently developed frontal cortex line rotational alignment technique is the same or better than the other two rotational alignment techniques in terms of biomechanics. This finding can be, however, dependent on the contact characteristics between the baseplate and the proximal tibia. That is, it is indicated that the optimum baseplate rotational arrangement technique in terms of reducing the incidence of TKA mechanical failure can be achieved by adjusting the characteristics of contact between the baseplate and the proximal tibia.

## 1. Introduction

Total knee arthroplasty (TKA) is a widely used orthopedic surgical approach for patients with severely painful joint diseases, such as degenerative arthritis, inflammatory arthritis, or avascular necrosis, and facilitates rapid resumption of high-level daily activities free of pain [1]. The survival rate of TKA is 90%–94% after 15- to 23-year follow-up [2]. However, Sharkey et al. [3] reported TKA failures due to loosening of the prosthesis, wear, infection, instability, pain, osteolysis, malalignment, or malposition.

TKA failures can be divided into biological failures and mechanical failures [4, 5]. Biological failures may be caused by infection, necrosis, or osteolysis, which are highly correlated with the presence of underlying disease, obesity, or postoperative infection [4]. Mechanical failures, which include failures not categorized as biological, are associated with mechanical factors such as prosthesis size, type, or arrangement, or unacceptable loads on the joint above the allowable limit resulting from excessive activity of the patient [2, 3]. Specifically, mechanical failures improve more readily and rapidly than biological failures. For this reason, researchers have sought to reduce the incidence of mechanical failures, particularly by developing new TKA designs to meet the joint characteristics of individual patients or by developing new surgical/procedural techniques and instruments to ensure accurate arrangement of TKA components; all of these techniques aim to improve the survival rate of TKA [6–15]. Baldini et al. [7] and Kim et al. [16] suggested a new baseplate rotational arrangement technique for TKA components based on the anterior tibial surface curvature, which simplifies the surgical procedure and enables more accurate rotational arrangement of the baseplate than conventional rotational arrangement techniques [7]. However, previous studies regarding baseplate rotational arrangement techniques used computed tomography (CT) to evaluate whether the intended baseplate arrangement had been achieved. In contrast, few studies have assessed the biomechanical characteristics (stress, strain, and micromotion) and clinical efficacy of baseplate rotational arrangement techniques. In particular, malrotation arrangement of the baseplate reportedly results in excessive stress on the proximal tibia under the baseplate, possibly causing tibia fracture, damaged bone cement, or tibial osteolysis [5, 7]. Thus, it is essential to evaluate the biomechanical characteristics and clinical efficacy of the suggested baseplate rotational arrangement techniques prior to their application in clinical practice.

This study involved evaluation of the biomechanical characteristics of TKA techniques according to baseplate rotational arrangements, including changes in principal stress within the proximal tibia, the strain on the cortical bone of the proximal tibia immediately below the baseplate, and peaked von Mises stress (PVMS) at the bone cement. Furthermore, we assessed the biomechanical factor important for the development of baseplate rotational arrangement techniques, with the aim of suggesting an approach to determining the optimal baseplate rotational arrangement technique to reduce the incidence of TKA mechanical failure. Our hypothesis was that the contact characteristics between the baseplate and the proximal tibia are important factors in the development of the rotational alignment technique of the base plate.

## 2. Materials and Methods

### 2.1. Finite Element (FE) Model Development

#### 2.1.1. Proximal Tibia and TKA Components

**Figure 1:**
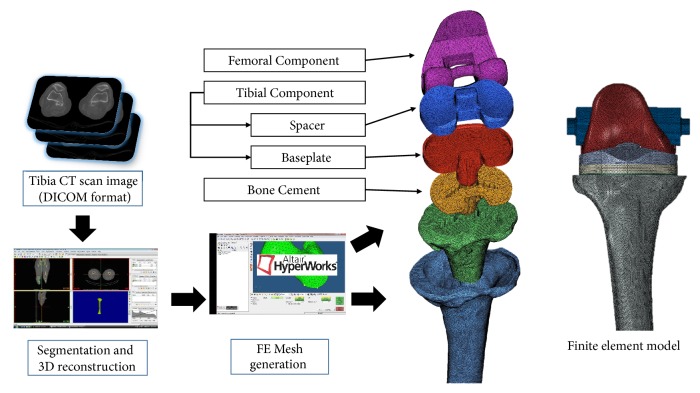
Finite element model of a proximal tibia with TKA.

A three-dimensional proximal tibia model for an FE model was established using Mimics 14.0 (Materialize, Leuven, Belgium) and CT scans (0.1-mm slice thickness using a 64-channel CT scanner [Somatom Sensation 64, Siemens Healthcare, Germany]) of the right tibia in a patient diagnosed with arthritis (Figure 1). Following Seoul National University Hospital approval, CT scans were accessed and treated by a physician (Professor Myung Chul Lee: One of Authors). To insert a baseplate into the tibia model, the proximal tibia was resected according to the TKA general surgical guidelines [17]. A tibial resection was made 2 mm below the medial articular surface of the tibia, perpendicular to the mechanical axis connecting the center of the femoral head to the center of the talus at the ankle joint [18], with an additional resection at 3° of posterior tilt of the baseplate. FE models of TKA femoral and tibial components were developed using LOSPA (Size #4; Corentec Corp. Republic of Korea) (Figure 1). The LOSPA considered in the current study was developed by adjusting TKA component design factors, such as anterior flange roundness, femoral spherical condyle, bearing surface contour, and patellofemoral surface, to improve knee joint stability [19, 20]. The proximal tibia and TKA component FE models were developed based on a three-dimensional model using Hyperworks 12.0 (Altair, USA). At this stage, the FE models used tetrahedron elements consisting of four nodes (C3D4); the material properties of the FE models are presented in Table 1 [21].

#### 2.1.2. Insertion, Alignment, and Arrangement of TKA Components

**Figure 2:**
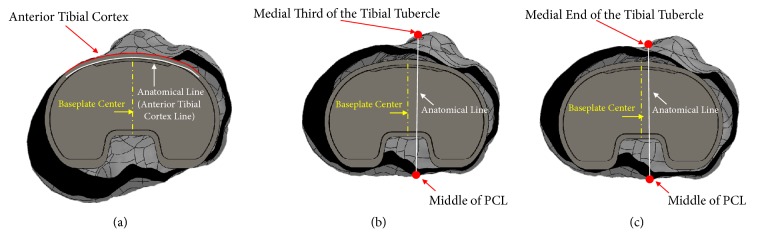
The (a) anterior cortex line, (b) tibial tuberosity one-third line, and (c) tibial tuberosity end line baseplate rotational arrangement techniques.

To describe the tibia with the artificial knee joint inserted, the TKA component FE model was inserted into the previously embodied tibial FE model (Figure 1) [19]. The baseplate was first aligned along the mechanical axis connecting the center of the head of the femur with the center of the ankle joint, and subsequently an anatomical line connecting the anterior with the posterior tibia was set using the bony landmarks of the proximal tibia (anterior tibial surface curvature, medial third of the tibial tubercle, and medial end of the tibial tubercle), followed by determining the rotational arrangement (orientation) of the baseplate based upon them and then performing the arrangement (Figure 2). To assess the effect of the baseplate arrangement technique on the biomechanical characteristics, three baseplate rotational arrangement techniques (anterior cortex line, tibial tuberosity one-third line, and tibial tuberosity end line) were considered. The first involved alignment of the anterior curvature of the baseplate with the anterior cortex line of the anterior tibial surface curvature of the resected surface of the proximal tibia [7, 16]. The second and third techniques are commonly used in clinical practice. The second technique aligns the center of the tibial baseplate tray with the line between the medial third of the tibial tubercle and the center of the posterior cruciate ligament (PCL) [7, 16, 22], and the third technique aligns the center of the tibial baseplate tray with the line making a connection between the medial end of the tibial tubercle and the center of the PCL [7, 16, 23]. Finally, bone cement was embodied in the plane of contact between the tibia and baseplate such that 1 mm of bone cement surrounded the entire plane of contact with the tibia to mimic an actual clinical setting. Here, it was assumed that the thickness of the bone cement is 1 mm based on a previous study and clinical data [24].

To establish the loading and contact conditions, the femoral component was placed perpendicular to the baseplate, whereas to mimic the actual contact conditions of the knee joint, the internal rotation angle of the femoral component ranged from 1.3° to 5.7° depending on the flexion of the knee joint [25]. Regarding medial and lateral tibial plateau loading conditions, five motions (bending the knee, sitting down, going up stairs, going down stairs, and standing up) frequently performed in daily life (activities of daily living [ADLs]) were selected from the Orthoload database (www.orthoload.com) [26]. The maximum joint reaction forces and their corresponding moments for the loading conditions considered were then selected and used for the FE analyses. The loading conditions covered for 0° to 90° flexion angles of the knee joint (Table 2). For the contact conditions, surface-to-surface contact conditions were adopted for each contact surface, and the following coefficients of friction between respective contacts were used: 1 between the tibia and bone cement, 0.4 between the bone cement and the baseplate, and 0.01 between the femoral component and the insert of tibial component. For the other contact surfaces, general contact conditions were assigned a friction coefficient of 0.4 [11–15, 24–27]. The boundary conditions were set to restrict the degrees of freedom of the distal tibia. The FE models were solved using Abaqus 6.12 (Dassault Systems, USA) to perform FE analyses.

### 2.2. Actual Mechanical Test Configuration for FE Model Validation

**Figure 3:**
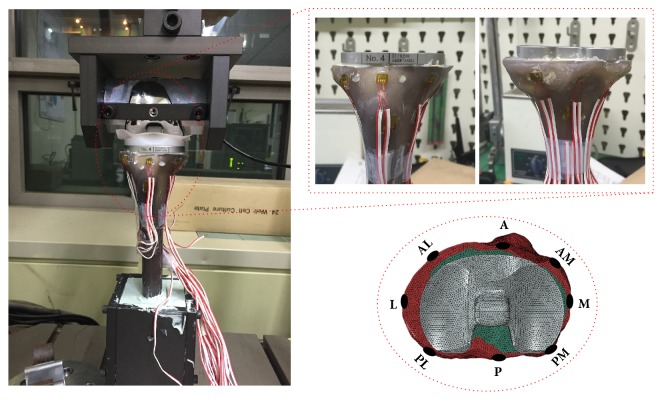
Mechanical test configuration for FE model validation. A, anterior; M, medial; L, lateral; P, posterior; AM, anterior-medial; AL, anterior-lateral; PM, posterior-medial; and PL, posterior-lateral region.

**Figure 4:**
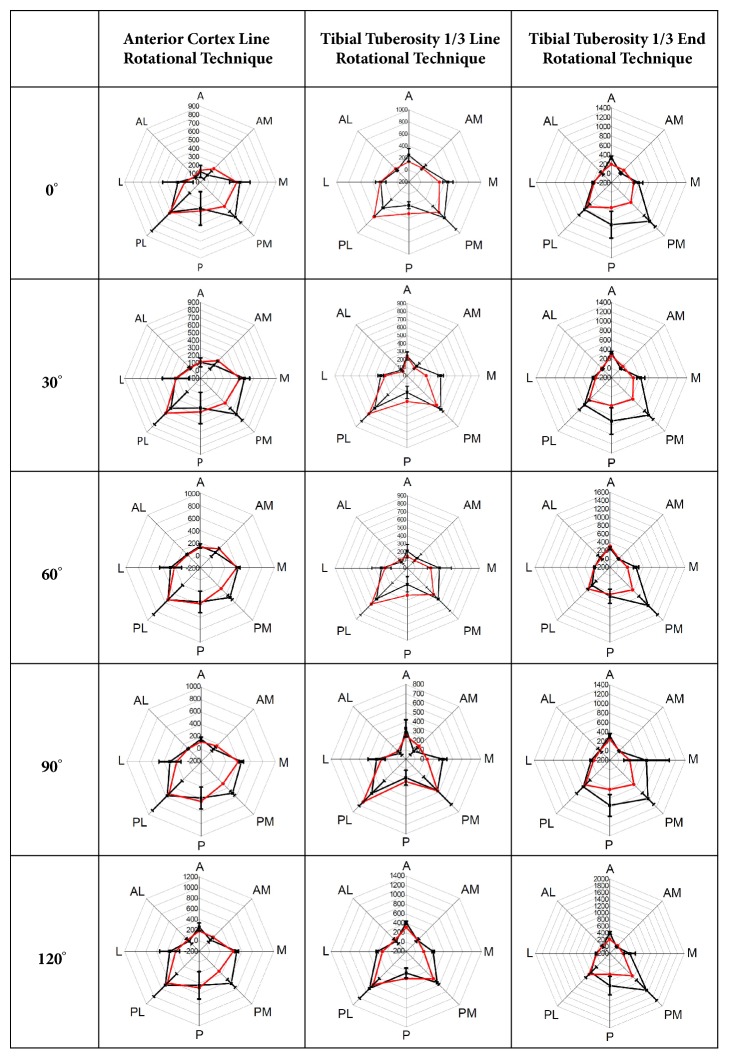
Results of the FE model validation (comparison of the strains from the FE models with those obtained from mechanical tests). A, anterior; M, medial; L, lateral; P, posterior; AM, anterior-medial; AL, anterior-lateral; PM, posterior-medial; and PL, posterior-lateral regions.

Mechanical tests based on the ASTM F1800 standard to validate the FE models were performed using a universal testing machine (Instron 8872; Instron Inc., USA) (Figure 3). The composite tibiae with primary TKA and the three baseplate rotational arrangement techniques were used for the mechanical tests (n = 18 total, n = 6 for each rotational arrangement). The procedures for resection of the composite tibia for the primary TKA and the insertion, alignment, and arrangement of the primary TKA components were as used for development of the FE models. The composite tibia with primary TKA and the femoral components were mounted on a customized jig attached to a universal testing machine. The customized jig was designed to represent knee flexion angles of 0° to 120° considering the rollback phenomenon. Eight strain gauges (Half-Bridge Type, CAS Corp., Republic of Korea) were attached to the surface of the proximal tibia near the resected surface. A data acquisition system (NI-9237 and CDAQ-9178; National Instrument, USA) was used to gather data from the strain gauges. A vertical load of 2,100 N (3 × 70 kg BW) through the femoral component was applied to the composite tibia with the primary TKA. The medial and lateral condyle force ratio (6:4) was obtained from a previous study [20]. FE analyses for validation were performed under the same loading and boundary conditions used in the mechanical tests using Abaqus 6.12 (Dassault Systems, USA). The FE models were validated by comparing the strains obtained from the strain gauges with those from the FE analyses. Eight regions of interest (ROIs), which were located at the same anatomical regions (strain gauge attachment locations) used in the mechanical tests, were used to compare the strain values obtained from the mechanical tests with those from the FE analyses (Figure 4). Additionally, convergence test of the FE model was performed to identify the influence of the number of elements on the results of FE model. The number of elements for the original FE model was ±10% decreased (coarsest mesh model) and increased (finest mesh model). The convergence test showed that the differences of the FE results were generally below 2% approximately in ±10% changes of the number of elements of the original FE model. This fact may indicate that the FE model used in the current study is not sensitive to the grid refinement of the mesh.

### 2.3. Data Analyses

To evaluate differences in biomechanical characteristics according to baseplate rotational arrangement technique, the principal stress flow within the proximal tibia, the strain on the cortical bone of the proximal tibia immediately below the baseplate, and the PVMS within the bone cement were assessed. First, to characterize stress transfer in the tibia in motions associated with ADLs according to the baseplate rotational arrangement technique, the principal stress flow inside the tibia was analyzed. Second, to predict the likelihood of tibial bone resorption, the strain on the cortical bone of the proximal tibia immediately below the baseplate was characterized. The likelihood of bone resorption was analyzed by comparing the critical bone damage strain (≤ 50 *µ*strain) reported previously with the strain results from the FE analyses [28]. Finally, the possibility of osteolysis in the tibia was analyzed by predicting the likelihood of bone cement failure by comparing the PVMS inside the bone cement calculated by FE analysis with the yield strength (21 MPa) of the bone cement [4].

## 3. Results

### 3.1. FE Model Accuracy

The strain results from the FE analyses and those from mechanical tests are shown in Figure 4. Compared to the latter, the former exhibited average differences of approximately 5.3 ± 5.6%. The values were almost identical in most proximal tibia regions, whereas significant differences of approximately 10.7%–17.7% were found in the medial and posterior-medial regions.

### 3.2. Principal Stress Flow within Tibial Cortical Bone

**Figure 5:**
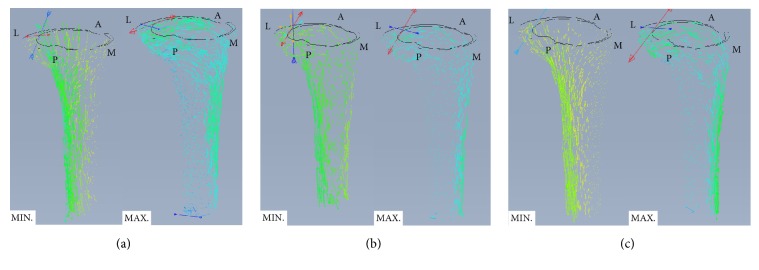
Representative maximum and medium principal stress flows (under loading conditions corresponding to the ADL of descending stairs) for the (a) anterior cortex line, (b) tibial tuberosity one-third line, and (c) tibial tuberosity end line rotational arrangement techniques (A, anterior; M, medial, L, lateral; and P, posterior regions).

Representative principal stress flow results within the tibial cortical bone according to baseplate rotational arrangement technique are shown in Figure 5. Slight differences in the maximum principal stress flow from the proximal tibia to the distal tibia were observed among the baseplate rotational arrangement techniques. For the tibial tuberosity one-third line and tibial tuberosity end line rotational arrangement techniques, the maximum principal stresses generally flowed from the medial region of the proximal tibia to the anterior region of the distal tibia for the loading conditions evaluated. In contrast, for the anterior cortex line rotational arrangement technique, the maximum principal stresses generally flowed from the medial region of the proximal tibia to the anterior and anterior-lateral regions of the distal tibia. The minimum principal stresses flowed mainly to the medial region irrespective of the baseplate rotational arrangement technique used.

### 3.3. Strain on Cortical Bone of the Proximal Tibia below the Baseplate

**Figure 6:**
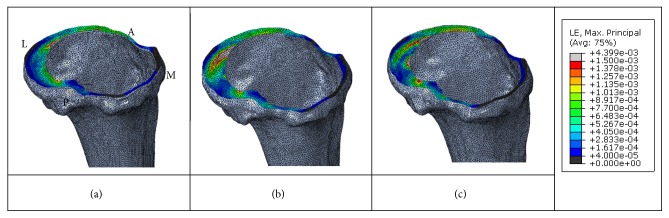
Representative strain distribution on the cortical bone of the proximal tibia immediately below the TKA baseplate (under loading conditions corresponding to the ADL of descending stairs) for the (a) anterior cortex line, (b) tibial tuberosity one-third line, and (c) tibial tuberosity end line rotational arrangement techniques (A, anterior; M, medial, L, lateral; and P, posterior regions).

Representative strain distributions on the cortical bone of the proximal tibia immediately below the baseplate according to the baseplate rotational arrangement technique are shown in Figure 6. Overall, the strains less than the critical bone damage strain (≤ 50 *µ*strain), which may affect bone remodeling, were the most widely distributed on the proximal tibia below the baseplate for the anterior cortex line rotational arrangement technique. The tibial tuberosity one-third line rotational arrangement technique resulted in less distributed strains, and the tibial tuberosity end line rotational arrangement technique exhibited the least distributed strains. For the tibial tuberosity one-third line rotational arrangement technique, strains ≤ 50 *µ*strain accounted for approximately 2.2%–11.3% in ADLs with high loading conditions. For the tibial tuberosity end line rotational arrangement and anterior cortex line rotational arrangement techniques, strains ≤50 *µ*strain accounted for approximately 2.3%–13.3% and 10.6%–16.6%, respectively, in ADLs with high loading conditions.

### 3.4. Peak Von Mises Stress within Bone Cement

Peak von Mises stresses (PVMSs) within the bone cement are summarized in Table 3. PVMSs greater than the yield strength (21 MPa) of the bone cement were generally concentrated in the medial region of the proximal tibia. For the anterior cortex line rotational arrangement technique, PVMSs within the bone cement ranged from 11.6 to 21.7 MPa in ADLs with high loading conditions. For the tibial tuberosity end line and one-third line rotational arrangement techniques, PVMSs ranged from 13.5 to 26.7 MPa and 19.4 to 29.2 MPa, respectively, in ADLs with high loading conditions.

## 4. Discussion and Conclusion

The accuracy of the FE models used in this study was high (94.7 ± 5.6%), although it was somewhat low (82.3%–89.3%) in the medial and posterior-medial regions of the proximal tibia. This low accuracy is likely due to differences between the isotropic material properties input into the FE model of linear elastic element and composite material actually used in the mechanical test and/or to the erroneous attachment of a strain sensor owing to the morphological specificity of the posterior region of the proximal tibia. In conclusion, our results may suggest that the FE models developed in this study show high validity.

The principal stress on the tibia was generally similar in the proximal region irrespective of the baseplate rotational arrangement technique used but differed in the distal region. Common to all baseplate rotational arrangement techniques, principal stress was higher and lower in the medial and lateral regions, respectively, of the proximal tibia. This finding is likely due to differences in the loading characteristics of the medial and lateral condyle of the tibia. By contrast, differences in the principal stress toward and in the distal tibia may result from the effect on baseplate location of the baseplate rotational arrangement technique used. Specifically, as the anterior cortex line rotational arrangement technique aligns the anterior curvature of the baseplate with the anterior cortex line of the resected surface of the proximal tibia, the baseplate is located anteriorly, unlike the tibial tuberosity one-third line and tibial tuberosity end line rotational arrangement techniques. Consequently, it appears that as the load contact location on the medial condyle moves to the anterior region, a greater bending moment may occur toward the anterior on the sagittal plane; thus, principal stress is exerted mainly on the anterior region of the distal tibia. In addition, this pattern of principal stress in the proximal and distal regions of the tibia may affect the strain in the tibia and the percentage of the critical bone damage strain (≤ 50 *µ*strain). In other words, critical bone damage strain occurred in the anterior-lateral, lateral, and posterior-lateral regions of the proximal tibia, which had low principal stress. This is consistent with the findings of Cawley et al. [29] and Innocenti et al. [5].

The critical bone damage strain ratio was the highest with the anterior cortex line rotational arrangement technique, followed by the tibial tuberosity one-third line rotational arrangement technique and the tibial tuberosity end line rotational arrangement technique. This finding (i.e., differences in the ratio of the critical bone damage strain among the baseplate rotational arrangement techniques) may be attributable to the difference in the characteristics of contact between the baseplate and the cortical bone of the proximal tibia. The anterior cortex line rotational arrangement technique exerts lower contact pressure on the cortical bone, resulting in a higher ratio of critical bone damage strain due to the large contact area between the baseplate and the cortical bone in the posterior-lateral region of the proximal tibia. In contrast, the tibial tuberosity one-third line and tibial tuberosity end line rotational arrangement techniques exert higher contact pressure on the cortical bone, leading to a lower ratio of the critical bone damage strain due to the smaller area of contact between the baseplate and the cortical bone in the posterior-lateral region of the proximal tibia, and because the contact involves only part of the cortical bone. These results suggest that characteristics of contact between the baseplate and the cortical bone affect the ratio of critical bone damage strains on the cortical bone of the proximal tibia. Therefore, the optimal baseplate rotational arrangement should be determined by adjusting the contact area. In conclusion, although diverse biological and mechanical factors influence bone resorption [4], the critical bone damage strain ratio suggests that the anterior cortex line rotational arrangement technique is most likely to facilitate bone resorption. However, because the critical bone damage strain ratio is influenced by the direction and magnitude of physiological loads on the tibia, this method may be unsuitable for predicting the possibility of bone resorption. For example, if the physiological load magnitude increases (e.g., the intensity of ADLs increases), the critical bone damage strain ratio will decrease irrespective of the baseplate rotational arrangement technique, which reduces the likelihood of bone resorption. Therefore, because diverse biological and mechanical factors related to the direction and magnitude of physiological load influence bone resorption, the ability to predict bone resorption based on the critical bone damage strain ratio is limited. However, our findings suggest that, for the same loading conditions (i.e., direction and magnitude of physiological load), the anterior cortex line rotational arrangement technique results in a higher critical bone damage strain ratio on the cortical bone of the proximal tibia than the other two techniques.

The bone cement PVMS results suggest the likelihood of bone cement failure. PVMSs exceeding the yield strength of the bone cement were the highest for the tibial tuberosity one-third line rotational arrangement technique, followed by the tibial tuberosity end line rotational arrangement technique and the anterior cortex line rotational arrangement technique. PVMSs were generally detected in the medial region of the bone cement. This finding is likely due to differences in the loading applied to the medial and lateral condyle of the tibia. In addition, the PVMS results are likely attributable to differences in the characteristics of the contact between the baseplate and the cortical bone of the proximal tibia. The characteristics of contact of the anterior cortex line rotational arrangement technique, in which the baseplate contacts the entire medial region of the cortical bone of the proximal tibia, led to the lowest frequency of PVMSs greater than the yield strength. In contrast, for the tibial tuberosity one-third line and tibial tuberosity end line rotational arrangement techniques, the baseplate contacts only part of the medial region of the cortical bone of the proximal tibia; therefore, the frequency of PVMSs greater than the yield strength was higher. Specifically, for the tibial tuberosity one-third line rotational arrangement technique (which has the smallest contact area), the frequency of PVMSs greater than the yield strength was the highest. These findings suggest that the contact area between the baseplate and the cortical bone of the proximal tibia influences the frequency of PVMSs greater than the yield strength, and the optimal baseplate rotational arrangement can be determined by adjusting the contact area. Bone cement failure generates bone cement debris (fragments), resulting in inflammation and osteolysis in the tibia [4]. According to our results, the risk of osteolysis in the medial region of the proximal tibia is higher, which is consistent with clinical outcomes [30]. Additionally, the anterior cortex line rotational arrangement technique may be associated with a lower risk of osteolysis due to bone cement failure. However, the ability of our results to predict the risk of osteolysis was limited because PVMSs within bone cement may also be affected by the direction and magnitude of the load applied to TKA. However, based on our results, the anterior cortex line rotational arrangement technique is associated with a lower frequency of PVMSs greater than the yield strength of bone cement than the other two techniques, leading to a reduced risk of bone cement failure and osteolysis. In addition, application of a greater physiological load to TKA will increase the frequency of PVMSs greater than the yield strength and reduce the critical bone damage strain ratio on the cortical bone. Therefore, the anterior cortex line rotational arrangement technique may be associated with a lower frequency of PVMSs greater than the yield strength and a reduced likelihood of bone resorption and osteolysis.

This study evaluated three baseplate rotational arrangement techniques commonly used in clinical practice in terms of their biomechanical characteristics and identified the biomechanical parameters that should be considered in the development of baseplate rotational arrangement techniques. We suggest an approach to determining the optimal baseplate rotational arrangement technique that increases the TKA survival rate by reducing the incidence of mechanical failure. However, this study had the following limitations: no consideration of the influence on alteration of baseplate rotational arrangement technique such as polyethylene wear of the tibial liner, related femoral rotation and patellar tracking and robustness of accuracy of alignment technique due to anatomical variation, incomplete analyses of the likelihood of bone resorption and osteolysis based on a single value (criterion) despite the influence of diverse biological and mechanical factors, and failure to evaluate clinical efficacy because of difficulty in performing longitudinal follow-up evaluations of patients within a short period. Nevertheless, to our knowledge, this study is the first to assess baseplate rotational arrangement techniques to identify the biomechanical parameters associated with a successful outcome. The results will facilitate development of a more effective baseplate rotational arrangement technique.

## Figures and Tables

**Table 1:** Material properties used in the FE model.

**Part of FE Model **	**Material Property**		
	**Elastic Modulus (GPa)**	**Poisson's Ratio**	**Density (g/cm** ^**3**^ **)**
**Cortical Bone**	17	0.36	1.64

**Cancellous Bone**	0.3	0.30	0.27

**Femoral Component**	200	0.33	8
**Tibia Component (Baseplate)**			

**Tibia Component (Spacer)**	0.9	0.46	0.94

**Bone Cement**	2.3	0.30	1.19

**Table 2:** Loading conditions corresponding to activities of daily living (ADLs).

**ADLs**	**Loading Parameters**								
	**Flexion ** **Angle (**°**)**	**Medial ** **Ratio **(%)	**Lateral ** **Ratio **(%)	**Medial ** **Force (N)**	**Lateral ** **Force (N)**	**Total ** **Force (N)**	**Moment** ** X (Nm)**	**Moment** ** Y (Nm)**	**Moment** ** Z (Nm)**
**Knee Bend**	90	71.0	29.0	-1375.1	-560.5	-1935.6	7.5	-7.0	3.3
	0	59.5	40.5	-515.4	-351.0	-866.4	3.1	5.1	1.8

**Sit Down**	90	71.0	29.0	-1295.4	-528.0	-1823.4	10.3	-2.6	3.9
	30	66.7	33.3	-649.7	-324.8	-974.5	7.6	3.0	0.7

**Stair Down**	60	70.1	29.9	-1747.8	-744.4	-2492.2	13.3	9.5	5.2
	30	66.7	33.3	-1537.9	-769.0	-2306.9	18.2	22.5	0.9

**Stair Up**	60	70.1	29.9	-1566.1	-667.1	-2233.2	10.0	13.8	1.4
	30	66.7	33.3	-223.5	-111.7	-335.2	1.8	-1.8	0.2

**Stand Up**	90	71.0	29.0	-1464.1	-596.7	-2060.8	9.4	-7.4	4.1
	0	59.5	40.5	501.0	-341.3	-842.3	3.9	1.9	2.0

**Table 3:** Peak von Mises stresses (PVMSs) within bone cement according to baseplate rotational arrangement technique.

**ADLs**	**Flexion ** **Angle (**°**)**	**PVMS (MPa)**		
		**Anterior ** **Cortex Line**	**Tibia Tuberosity ** **1/3 Line**	**Tibia Tuberosity ** **End Line**
**Knee Bend**	00	7.2	7.2	9.2
	90	19.9	20.3	14.4

**Stand Up**	00	7.1	6.9	9.2
	90	19.9	21.2	15.4

**Sit Down**	30	7.7	10.5	12.8
	90	21.7	19.4	13.5

**Stair Down**	30	11.7	27.3	26.7
	60	17.9	29.2	22.5

**Stair Up**	30	9.1	6.2	19.5
	60	16.2	25.3	20.3

## Data Availability

Data were included in this article.
